# 24 Hours: A Case of Multiorgan Failure Associated With COVID-19

**DOI:** 10.7759/cureus.10149

**Published:** 2020-08-31

**Authors:** Diana Espinoza, Raul Rodriguez, Andrew Kowalski

**Affiliations:** 1 Internal Medicine, MacNeal Hospital, Berwyn, USA; 2 Nephrology, MacNeal Hospital, Berwyn, USA

**Keywords:** covid-19, renal failure, risk factors, cytokine storm, mortality, acidosis, hemodialysis

## Abstract

Coronavirus has caused thousands of deaths due to several mechanisms of injury including acute kidney injury (AKI). Most of the patients have a fast progression of the disease leading to death in the second week of hospital admission, however, here we have a case of a 58-year-old female who died in less than 24 hours of admission due to severe metabolic acidosis, acute respiratory distress syndrome (ARDS) and renal failure.

## Introduction

Since its outbreak, coronavirus disease 2019 (COVID-19) has caused more than 600,000 deaths worldwide. The progression of the disease can be fast depending on comorbidities and organ dysfunction, it can take around two weeks from the onset of symptoms to death [1,2]. In critically ill patients, it is theorized that a cytokine storm and inflammatory response are associated with poor prognosis and mortality [2]. Severe acute respiratory syndrome coronavirus 2 (SARS-Cov-2) targets mainly the lungs causing fatal pneumonia, acute respiratory distress syndrome (ARDS) and respiratory failure; however, there is evidence that these patients present with additional multiorgan damage including cardiovascular, renal, gastrointestinal, neurological, and other complications [1]. Here, we present a case of a patient who expired in less than 24 hours of admission with rapid progression to multiple organ failure.

## Case presentation

A 58-year-old female with a past medical history of hypertension and obesity presented to the emergency department with shortness of breath and subjective fever for one day.

**Figure 1:**
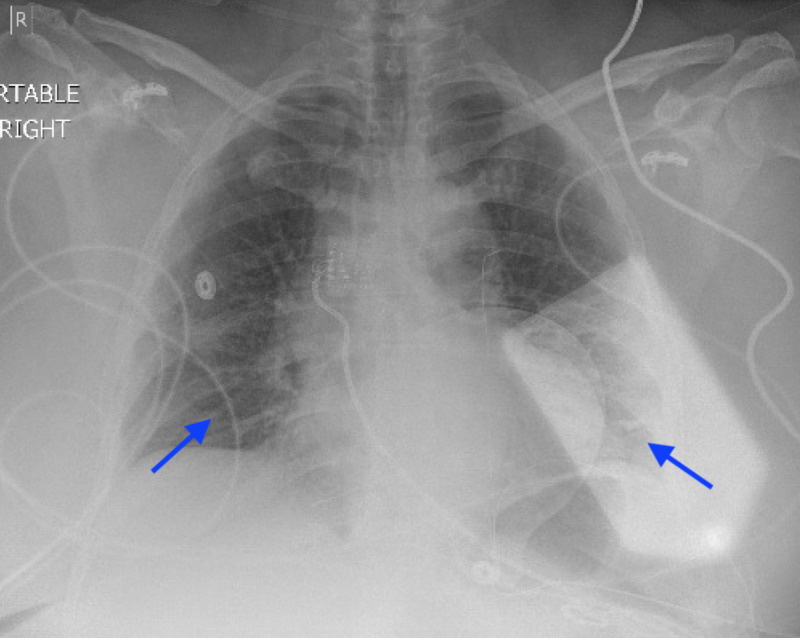
Portable chest X-ray Portable chest X-ray showing bilateral patchy airspace opacities.

**Figure 2:**
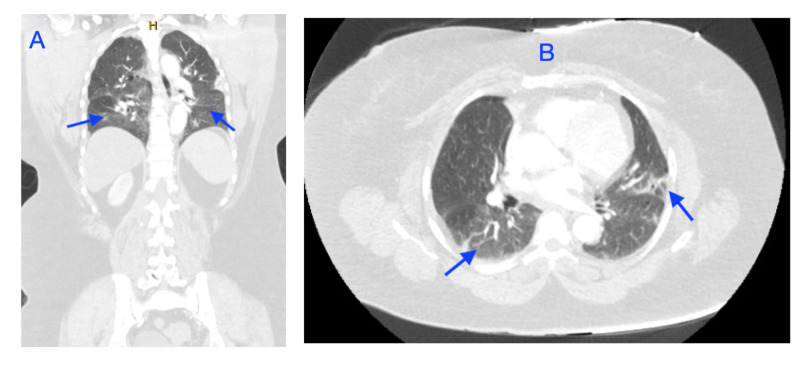
Computer tomography of the chest, abdomen and pelvis with IV contrast CT chest transversal cut showing bilateral ground-glass opacities (A) and CT chest coronal cut showing bilateral ground-glass opacities (B).

Hour 1-3: Upon arrival, vital signs: temperature 97.9 F^o^, blood pressure 135/98 mmHg, respiratory rate 35 per minute, heart rate 89 beats per minute and oxygen saturation 97% on room air. The nasopharyngeal sample for COVID-19 polymerase chain reaction (PCR) testing was collected and initial blood work revealed multiple metabolic derangements including elevated potassium 5.4 mmol/L, creatinine 4.84 mg/dl, glucose 284 mg/dl, Beta-hydroxybutyrate 0.9 mmol/L, C-reactive protein 7.4 mg/dl, alkaline phosphatase 121 U/L, aspartate aminotransferase 43 U/L, white blood cells 12.4 K/UL, lactic acid 4.9 mmol/L, D-Dimer 2.86 mg/L and severe acidosis with bicarbonate of 12 mmol/L, anion gap 23, arterial blood gas pH 7.19, partial pressure of carbon dioxide (PaCO2) 33, partial pressure of oxygen (PaO2) 121 on two liters via nasal cannula (NC); normal thyroid function and normal B-Natriuretic peptide. Chest X-ray (CXR) revealed patchy airspace opacities bilaterally (Figure 1). Computer tomography (CT) of the head, abdomen and pelvis were negative for acute process and CT of the chest showed bilateral ground-glass opacities otherwise unremarkable (Figure 2). Normal urine analysis and negative urinary antigens.

Hour 4-6: The patient was admitted to the Intensive Care Unit (ICU) due to severe sepsis likely secondary to viral pneumonia with worsening hypoxia, saturating 90% on six liters via nasal cannula (NC); oxygen requirements increased progressively, she developed altered mental status, bradycardia with a heart rate of 40 beats per minute, and hypotension not responsive to fluid boluses requiring emergent intubation as well as pressor support.

**Figure 3:**
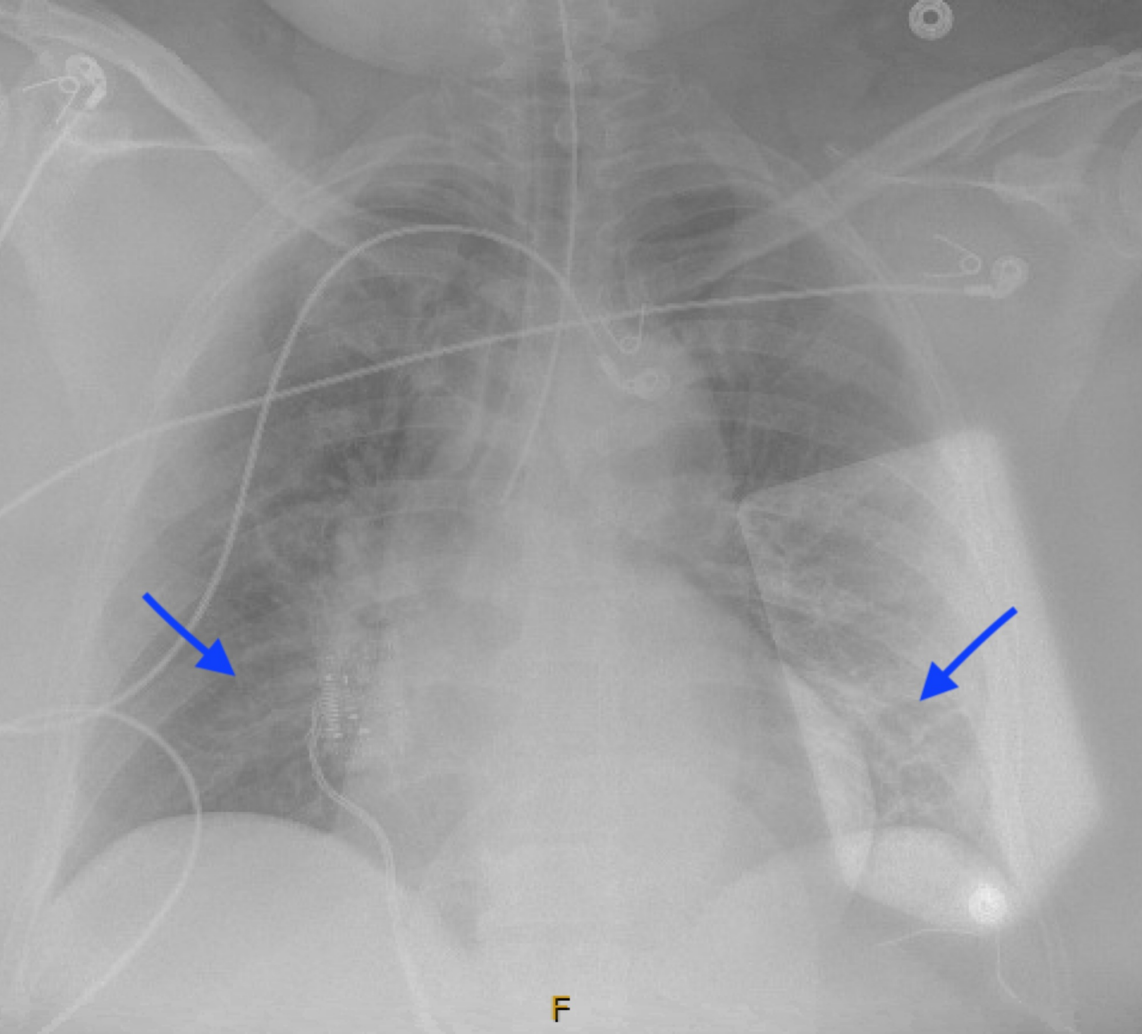
Portable chest X-ray Chest X-ray seven hours after admission, showing worsening bilateral patchy infiltrates.

Hour 7-19: Her urine output declined, she developed electrolyte imbalance and severe acidosis which prompted renal replacement therapy. A right internal jugular triple lumen catheter for hemodialysis was placed; however, hemodialysis was held due to severe hemodynamic instability. The patient required increasing vasopressor support. A repeat chest X-ray (CXR) showed worsening bilateral patchy infiltrates (Figure 3). Further blood work was significant for refractory hyperkalemia 6.6 mmol/L, worsening renal function with creatinine of 5.02 mg/dl and severe metabolic acidosis with a lactic acid of 15.7 mmol/L and an arterial blood gas showing: pH 6.83, PaCO_2 _35, PaO_2_ 128, bicarbonate < 4 mmol/L (Table 1). There was no improvement despite a bicarbonate drip. She also developed acute liver failure followed by acute liver shock and negative workup for disseminated intravascular coagulation (DIC). Finally, after 17 hours of admission, the result came back positive for COVID-19.

**Table 1:** Laboratory results

	Hour 1	Hour 6	Hour 12	Hour 18	Reference range
White blood cells	12.4	17.6	16.6	13.2	4.8-10.8 K/UL
Red blood cells	6.55	6.05	4.66	3.29	4.60-6.20 M/UL
Hemoglobin	18.4	17.2	13.1	9.3	13.0-17.5 gm/dl
Platelets	309	257	169	79	150-400 K/UL
Lymphocytes	9	9	12	10	1.0-4.0 K/ mm3
Sodium	133	127	138	138	136-144 mmol/L
Potassium	5.4	8.2	6.6	7.0	3.3-5.1 mmol/L
Chloride	98	101	108	11	95-111 mmol/L
Bicarbonate	12	6	<4	<4	20-32 mmol/L
Blood urea nitrogen	74	74	64	53	6-21 mg/dl
Creatinine	4.84	4.61	5.02	4.01	0.60-1.40 mg/dl
Calcium	8.1	7.3	5.5	3.7	8.5-10.5 mg/dl
Glucose	284	338	115	110	70-100 mg/dl
Albumin	3.0	-	1.0	0.6	3.6-5.0 gm/dl
Total Protein	6.8	-	3.3	1.9	6.5-8.3 gm/dl
Alkaline phosphatase	121	-	93	121	30-110 U/L
Alanine aminotransferase	26	-	719	1474	10-45 U/L
Aspartate aminotransferase	43	-	1336	3861	14-25 U/L
Total bilirubin	0.3	-	0.4	0.3	0.2-1.4 mg/dl
Anion Gap	23	20	23	23	4-16
Lactic Acid	4.9	9.5	15.7	-	0.0-2.0 mmol/L
C-Reactive protein	7.4	-	-	-	0.0-1.0 mg/dl
Ferritin	-	17221	-	71927	21-267 ng/ml
Lactate dehydrogenase	-	935	-	6471	108-212 U/L
D-Dimer	-	2.49	-	2.86	0.17-0.57 mg/L
Troponin (POC)	0.01	-	-	-	0.00-0.08 ng/ml
Prothrombin time	11.5	11.5	-	-	9.1-11.2 sec
International normalized ratio	1.1	1.1	-	-	0.8-1.2
Partial thromboplastin time	-	37.5	-	-	22.5-29.9 sec
Arterial blood gas	7.19/33/121	6.83/58/310	6.86/38/164	6.83/35/128	pH/PaCO2/PaO2

Hour 20: The patient went into cardiac arrest with pulseless electrical activity; advanced cardiovascular life support (ACLS) maneuvers were performed; however, the return of spontaneous circulation (ROSC) was not achieved and the patient was pronounced dead.

## Discussion

Given the new COVID-19 pandemic, multiple retrospective studies around the world have been trying to identify predictive risk factors associated with disease severity and mortality. Of these, a retrospective cohort study in Wuhan found that age, D dimer > 1 ug/ml and high sequential organ failure assessment (SOFA) on admission increased the odds of deaths; also the majority of non- survivors developed septic shock, acute cardiac injury, coagulopathy, acute kidney injury (AKI) and severe acidosis [3,4]. The decompensation timeline varies depending on specific patient characteristics; on average most of the patients developed sepsis within 10 days of hospitalization and died by day 18 [3]. Critically ill patients who had a rapid decompensation initially presented to the health care services with mild symptoms like cough, fever, dyspnea followed by a rapid deterioration in ARDS and multiple organ dysfunction which could be explained by the cytokine storm [5]. Most of the reported deaths are secondary to respiratory failure with ARDS followed by cardiac complications, AKI, and liver shock [6]. 

Our patient clearly had a rapid onset of septic shock, acute renal failure with significant metabolic acidosis, ARDS and ischemic hepatitis, having a fatal outcome in less than 48 hours of symptomatology and less than 24 hours of admission, which is not the most common presentation. Rapid deterioration and critical outcomes have been described in the literature to be secondary to a rapid decline in renal function with the development of severe metabolic acidosis due to cytokine storm associated with COVID-19 [5]. This severe metabolic acidosis could be a consequence of the tissue hypoxia due to ARDS causing conversion of the pyruvate to lactic acid. Also, the extensive microvascular thrombosis well described now on COVID-19 patients could explain the severe organ dysfunction [6]. 

AKI has also been independently associated with inpatient mortality. According to a prospective cohort study in Wuhan by Cheng et al., Stage 3 AKI had a hazard ratio for inpatient mortality of 9.81 [7]. This was also suggested in a retrospective study by Pei et al., in which patients with renal involvement had higher mortality (11.2%) vs those without renal involvement (1.2%) [8]. AKI can be present in up to 19% of critically ill patients with COVID-19 and it usually develops within five to seven days of admission [9]. Considering that most of these patients develop septic shock the pathogenesis of renal dysfunction could be related to hypoperfusion itself, acute tubular necrosis (ATN), microvascular thrombosis or direct damage [10]. SARS-Cov-2 has a great affinity for angiotensin-converting enzyme (ACE) 2 receptors and some patients can have an upregulation of these receptors which could explain why some patients develop renal failure earlier and faster [10,11]. Su et al. performed post mortem kidney biopsy on patients who died with multiple organ dysfunction secondary to COVID-19 [12]. There were findings of diffuse proximal tubule injury, necrotic changes and obstruction of lumen capillaries under light microscopy and under electron microscopy, particles of coronavirus and antibody complexes in the tubular epithelium and podocytes were identified. In the urine common findings were proteinuria and viral ribonucleic acid (RNA) [8-12]. 

The treatment of critically ill patients with rapid progression to organ failure remains unclear. In the meantime, supportive and standard ICU protocols are followed which includes conservative fluid resuscitation and use of diuretics if needed [13]. Previously with severe acute respiratory syndrome (SARS) and Middle East respiratory syndrome coronavirus (MERS-CoV) infections, AKI was identified and continuous renal replacement therapy (CRRT) showed to improve the prognosis [11]. CRRT is the recommended modality in hemodynamically unstable COVID-19 patients; however, the different renal replacement therapy (RRT) modalities have not been compared on studies [13]; after seeing our case perhaps those patients should be started on renal replacement therapy as soon as intubation occurs if rapid decompensation is expected.

## Conclusions

AKI on these patients clearly is related to several mechanisms including systemic hypoxia, cytokine storm, abnormal coagulation and direct damage. The identification of predictors of severity and mortality could help physicians to adopt early therapeutic strategies, avoid the progression of the disease to multi-organ damage and eventually death. One of those strategies could be early CRRT. Our patient clearly had indications for it, however, given her rapid deterioration requiring pressor support with four medications, she would have been unable to tolerate hemodialysis, thus having a fatal outcome. Although additional interventions like hemodialysis, IL-6 inhibitors could have been considered in this patient, therapeutic benefit would be difficult to assess considering rapid deterioration of her clinical status.

## References

[1] Chen T, Wu D, Chen H (2020). Clinical characteristics of 113 deceased patients with coronavirus disease 2019: retrospective study. BMJ.

[2] Huang C, Wang Y, Li X (2020). Clinical features of patients infected with 2019 novel coronavirus in Wuhan, China. Lancet.

[3] Zhou F, Yu T, Du R (2020). Clinical course and risk factors for mortality of adult inpatients with COVID-19 in Wuhan, China: a retrospective cohort study.. Lancet.

[4] Hu B, Wang D, Hu C (2020). Clinical features of critically ill patients with COVID-19 infection in China. Res Square.

[5] Chhetri S, Khamis F, Pandak N, Al Khalili H, Said E, Petersen E (2020). A fatal case of COVID-19 due to metabolic acidosis following dysregulate inflammatory response (cytokine storm). IDCases.

[6] Giannis D, Ziogas IA, Gianni P (2020). Coagulation disorders in coronavirus infected patients: COVID-19, SARS-CoV-1, MERS-CoV and lessons from the past. J Clin Virol.

[7] Cheng Y, Luo R, Wang K (2020). Kidney disease is associated with in-hospital death of patients with COVID-19. Kidney Int.

[8] Pei G, Zhang Z, Peng J (2020). Renal involvement and early prognosis in patients with COVID-19 pneumonia. J Am Soc Nephrol.

[9] Zaim S, Chong JH, Sankaranarayanan V, Harky A (2020). COVID-19 and multi-organ response. Curr Probl Cardiol.

[10] Durvasula R, Wellington T, McNamara E, Watnick S (2020). COVID-19 and kidney failure in the acute care setting: our experience from Seattle. Am J Kidney Dis.

[11] Naicker S, Yang C, Hwang S, Liu B, Chen J, Jha V (2020). The novel coronavirus 2019 epidemic and kidneys. Kidney Int.

[12] Su H, Yang M, Wan C (2020). Renal histopathological analysis of 26 postmortem findings of patients with COVID-19 in China. Kidney Int.

[13] Sise ME, Baggett MV, Shepard JO, Stevens JS, Rhee EP (2020). Case 17-2020: a 68-year-old man with COVID-19 and acute kidney injury. New Engl J Med.

